# Standardisierte Erfassung der gesundheitsbezogenen Lebensqualität bei Patienten mit Psoriasisarthritis: Validierung der deutschen Version des „Fragebogen zur Lebensqualität von Patienten mit Psoriasisarthritis (PsAQoL)“

**DOI:** 10.1007/s00393-020-00843-x

**Published:** 2020-08-03

**Authors:** U. Kiltz, I. Andreica, M. Igelmann, L. Kalthoff, D. Krause, E. Schmitz, S. P. McKenna, J. Braun

**Affiliations:** 1 Rheumazentrum Ruhrgebiet, Herne und Ruhr-Universität Bochum, Claudiusstr. 45, 44649 Herne, Deutschland; 2 Praxis für Rheumatologie, Bochum, Deutschland; 3 Privatärztliche Praxis für Immunologie, Rheumatologie, Osteologie, Bochum, Deutschland; 4 Rheumatologische Gemeinschaftspraxis, Gladbeck, Deutschland; 5 Praxis für Rheumatologie, Hattingen, Deutschland; 6 Galen Research Ltd. Manchester, Manchester, Vereinigtes Königreich

**Keywords:** Übersetzung, Feldtest, Krankheitsspezifisches Messinstrument, Psychometrische Eigenschaften, Psoriasis, Translation, Field test, Disease-specific measuring instrument, Psychometric properties, Psoriasis

## Abstract

**Einleitung:**

Die standardisierte Beurteilung der gesundheitsbezogenen Lebensqualität gewinnt in der Rheumatologie zunehmend an Bedeutung. Der englische Fragebogen „Psoriasis Arthritis Quality of Life Questionnaire (PsAQoL)“ ist ein krankheitsspezifisches Instrument zur Messung der Lebensqualität von Patienten mit Psoriasisarthritis (PsA). Ziel der vorliegenden Arbeit ist die Übersetzung des PsAQoL ins Deutsche und die Validierung der deutschen Version in einer aus der Routineversorgung rekrutierten Kohorte von PsA-Patienten.

**Methode:**

Die Übersetzung und Validierung des Fragebogens PsAQoL wurde in einem mehrstufigen Verfahren unter Beteiligung von betroffenen Patienten mit PsA durchgeführt. Nach Übersetzung des englischsprachigen Fragebogens wurde die deutsche Version in einem Feldtest überprüft. Die psychometrischen Merkmale des Fragebogens wurden anschließend in einer PsA-Kohorte aus der Routineversorgung untersucht. Neben der Konstrukt- und Gruppenvalidität wurden die Zuverlässigkeit des Fragebogens mittels Test/Retest-Reliabilität sowie die interne Konsistenz getestet. Die körperliche Funktionsfähigkeit wurde mit dem Health Assessment Questionnaire (HAQ) und Domänen der Lebensqualität mit dem Nottingham Health Profile (NHP) gemessen.

**Ergebnis:**

In einem Feldtest mit 10 Patienten erwies sich die deutsche Version des PsAQoL-Fragebogens als relevant, gut verständlich und durchführbar (Bearbeitungszeit: 4,7 ± 2,1 min). Insgesamt 126 Patienten (37,3 % männlich, Alter 55,6 ± 11,3 Jahre) wurden in die Validierungskohorte eingeschlossen. Der PsAQoL korrelierte moderat mit dem HAQ (r = 0,65) sowie moderat bis gut mit dem NHP (Subdomänen r = 0,58–0,75). Die interne Konsistenz war hoch (Cronbach’s α 0,92), und die Zuverlässigkeit bei Patienten mit stabilem Krankheitsverlauf war sehr gut (Spearman-Korrelationskoeffizient 0,94). Der PsAQoL kann zwischen unterschiedlichen Patientengruppen differenzieren.

**Zusammenfassung:**

Mit der deutschen Übersetzung des PsAQoLs steht ein valides und krankheitsspezifisches Messinstrument zur standardisierten Erfassung der gesundheitsbezogenen Lebensqualität bei Patienten mit PsA zur Verfügung. Die psychometrischen Eigenschaften der englischsprachigen Originalversion sind vergleichbar. Der deutsche PsAQoL kann damit für die klinische und wissenschaftliche Verwendung empfohlen werden.

## Abstract (en)

**Background:**

The standardized assessment of health-related quality of life is becoming increasingly more important. The English questionnaire on psoriatic arthritis quality of life (PsAQoL) is a disease-specific instrument for measuring the quality of life of patients with psoriatic arthritis (PsA). The aim of the present study was to translate the PsAQoL into German and to validate the German version in a cohort of PsA patients recruited from routine care.

**Method:**

The translation and validation of the PsAQoL questionnaire was carried out in a stepwise procedure involving affected patients with PsA. After translation of the original English questionnaire the German version was evaluated in a field test. The psychometric features of the questionnaire were then examined in a PsA cohort from routine care. In addition to the construct and group validity, the reliability of the questionnaire was tested using test-retest reliability and internal consistency. The physical functioning was measured with the health assessment questionnaire (HAQ) and domains of the quality of life with the Nottingham health profile (NHP).

**Results:**

In a field test with 10 patients the German version of the PsAQoL questionnaire proved to be relevant, easily understandable and feasible (processing time 4.7 ± 2.1 min). A total of 126 patients (37.3% male, age 55.6 ± 11.3 years) were included in the validation cohort. The PsAQoL showed moderate correlation with the HAQ (r = 0.65) and moderate to good correlation with the NHP (subdomains r = 0.58–0.75). The internal consistency was high (Cronbach’s α 0.92) and reliability in patients with stable disease course was very good (Spearman correlation coefficient 0.94). The PsAQoL can differentiate between different patient groups.

**Conclusion:**

The German translation of the PsAQoL provides a valid disease-specific instrument for the standardized assessment of health-related quality of life in patients with PsA. The psychometric characteristics of this questionnaire are comparable with the original English version. The German PsAQoL can therefore be recommended for clinical and scientific application.

Die Psoriasisarthritis (PsA) ist eine chronisch verlaufende entzündliche Systemerkrankung mit facettenreichen klinischen Manifestationen. Neben der muskuloskeletalen Beteiligung in Form von Arthritis, Enthesitis, Daktylitis und/oder axialen Manifestationen können eine Haut- und/oder Nagelbeteiligung sowie andere entzündliche Erkrankungen wie Uveitis oder eine chronisch entzündliche Darmerkrankung (CED) vorliegen [1, 2]. Entsprechend vielfältig präsentiert sich die klinische Symptomatik, die neben Schmerzen, Steifigkeit und Funktionsminderung der Gelenke von unterschiedlich stark ausgeprägten Hautmanifestationen geprägt ist. Insbesondere die variablen und teilweise entstellenden Hautmanifestationen tragen zum Auftreten von Schlafstörungen und psychischen Komorbiditäten, insbesondere von depressiven Erkrankungen, bei [3]. Darüber hinaus liegt eine Assoziation mit anderen Krankheitsentitäten wie dem Typ-2-Diabetes, der arteriellen Hypertonie, dem metabolischen Syndrom sowie einer Fettleber vor. Unbehandelt kann die periphere und axiale Gelenkbeteiligung zu einer Gelenkzerstörung bis hin zur Arthritis mutilans und dem Vollbild einer ankylosierenden Spondylitis führen [1, 2]. Das Fortschreiten der Gelenkzerstörung ist oft mit größeren Behinderungen verbunden, und die gesundheitsbezogene Lebensqualität (HRQoL) nimmt mit zunehmenden Funktionseinschränkungen ab [4].

Das Ausmaß der funktionellen und psychischen Beeinträchtigungen bei Patienten mit PsA ist denen mit rheumatoider Arthritis oder axialer Spondyloarthritis vergleichbar [5]. Dadurch kommt es bei PsA-Patienten zu stärkeren Beeinträchtigungen der Erwerbstätigkeit, zur häufigeren Inanspruchnahme von Gesundheitsdienstleistungen sowie mehr Krankenhausaufenthalten im Vergleich zu Patienten mit reinem Hautbefall [6].

Die HRQoL wird im Allgemeinen als mehrdimensionaler Begriff definiert, der die Bereiche körperliches Befinden (Allgemeinbefinden), psychische Verfassung (kognitive und emotionale Faktoren), soziale Einbindung (soziale Unterstützung und Integration), Lebenszufriedenheit und subjektive Gesundheit umfasst [7]. Die Erfassung der HRQoL mit validierten Fragebögen ermöglicht eine standardisierte Erfassung dieses multidimensionalen Konstrukts und beinhaltet daher die Erfassung der subjektiven Sichtweise des Patienten sowie auf Gruppenebene die Erfassung der Unterschiede zwischen verschiedenen Behandlungen. Häufig benutzte Instrumente zur Messung der HRQoL sind die generischen Messinstrumente Short Form-36 (SF-36), der European Quality of Life 5 Dimensions (EQ-5D) oder der Nottingham Health Profile(NHP)-Fragebogen [8–10]. In einem durch GRAPPA (Arbeitsgruppe Group for Research and Assessment of Psoriasis and Psoriatic Arthritis) und OMERACT (Outcome Measures in Rheumatology Clinical Trials) 2018 veröffentlichten systematischen Review zu psychometrischen Charakteristika der Patient Reported Outcomes (PROs) für PsA zeigte sich neben einer großen Heterogenität der Messinstrumente, dass die untersuchten generischen Fragebögen (SF36, HAQ und „Arthritis impact Measurement Social Support Scale“ [AIMS]) keine ausreichenden psychometrischen Qualitäten zur Messung der Lebensqualität bei Patienten mit PsA besitzen [11].

Krankheitsspezifische Instrumente zur Erfassung der HRQoL basieren auf der Überlegung, dass die grundlegenden Domänen der Lebensqualität innerhalb der Betroffenen vergleichbar sind [12]. Zu den krankheitsspezifischen HRQoL-Instrumenten bei PsA gehören PsAQoL und „Psoriatic Arthritis Impact of Disease-12“ (PsAID-12) [13, 14]. Dabei wurde der PsAQoL, basierend auf dem „Needs-based-Modell“, (bedarfsorientierte Lebensqualität) entwickelt. Das Modell basiert auf der Prämisse, dass die Lebensqualität abhängig ist von der Fähigkeit einer Person, ihre Bedürfnisse zu erfüllen. In dem Modell wird vorausgesetzt, dass die Lebensqualität am höchsten ist, wenn die menschlichen Bedürfnisse erfüllt werden, und am niedrigsten, wenn nur wenige Bedürfnisse erfüllt werden [15].

Für die PsA wurde, basierend auf dem Prinzip der bedarfsorientierten Lebensqualität, 2004 von McKenna et al. ein entsprechendes Instrument in Großbritannien entwickelt und validiert [14]. Der PsAQoL-Fragebogen wurde in mehrere Sprachen (Schwedisch, Niederländisch, Portugiesisch, Chinesisch und Englisch für Singapur, Griechisch) übersetzt und validiert, liegt aber noch nicht in einer Übersetzung für Deutschland vor [16–20].

Das Ziel des hier dargestellten Projektes ist die Übersetzung und Validierung der deutschen Übersetzung des PsAQoL-Fragebogens in einer Kohorte von Patienten mit PsA.

## Methoden

Die Übersetzung und Validierung des PsAQoL-Fragebogens wurden in einem mehrstufigen Verfahren unter Beteiligung von Patienten mit PsA durchgeführt: (I) Übersetzung des PsAQoL-Fragebogens, (II) Testung der Übersetzung in einem Feldtest und (III) Validierung der deutschen Übersetzung des PsAQoL in einer PsA-Kohorte aus der Routineversorgung.

Der PsAQoL-Fragebogen beinhaltet 20 Aussagen mit dichotomer Antwortoption („zutreffend“ und „nicht zutreffend“). Der Punktwert der einzelnen Antwortoptionen („zutreffend“ = 1 Punkt und „nicht zutreffend“ = 0 Punkte) ergibt den Summenwert. Der Summenwert des PsAQoL-Fragebogens umfasst den Wertebereich von 0 bis 20, wobei ein hoher Score schlechte Lebensqualität anzeigt.

### Übersetzung des PsAQoL-Fragebogens

Die englische Originalfassung des PsAQoL wurde mittels der Panel-Methode ins Deutsche übersetzt.

Die Panel-Methode sieht vor, dass neben einer Übersetzung durch zweisprachige Personen (Englisch/Deutsch) mit Deutsch als Muttersprache auch eine Testung auf Verständlichkeit der deutschen Übersetzung durch Laien erfolgt und anschließend evtl. eine Adaptation der Übersetzung erfolgen muss. Es sollte bei jeder Frage ein Konsens zwischen den zweisprachigen Experten und den Laien erzielt werden. Die Beteiligung von medizinischen Laien soll sicherstellen, dass die endgültige Version des Fragebogens laienverständlich ist und die Übersetzung dadurch eine breitere Akzeptanz aufweist. Laien aus dem Umfeld des Rheumazentrums Ruhrgebiet wurden eingeladen, an der Übersetzung des PsAQoL-Fragebogens ins Deutsche mitzuwirken.

### Testung der Übersetzung in einem Feldtest

Zur Überprüfung der Augenschein- und Inhaltsvalidität der deutschen Version wurden PsA-Patienten aus der Routineversorgung des Rheumazentrums Ruhrgebiet interviewt. Die Patienten haben die deutsche Übersetzung des PsAQoLs in Anwesenheit eines Untersuchers beantwortet und zu dem Inhalt des Fragebogens in einem strukturierten Interview Stellung bezogen. Es wurde Wert auf eine heterogene Stichprobe gelegt, sodass Patienten mit unterschiedlichem Alter, Geschlecht, Krankheitsmanifestation und Bildungsniveau eingeschlossen wurden, um die Verständlichkeit gewährleisten zu können. Patienten ohne Deutschkenntnisse wurden ausgeschlossen. Der Studienarzt dokumentierte soziodemografische Faktoren sowie klinische Charakteristika des Patienten und erfasste die Zeitdauer, die der Patient zur Beantwortung der Fragebögen benötigte. Die Reaktion des jeweiligen Patienten während der Beantwortung des PsAQoL-Fragebogens wurde beobachtet, und aufgetretene Verständnisschwierigkeiten wurden geklärt. Nachdem der PsAQoL vom Patienten selbstständig beantwortet worden war, fand ein qualitatives Interview zwischen Patienten und Untersucher hinsichtlich des Inhalts und der Länge des PsAQoL, der Angemessenheit, Verständlichkeit, Vollständigkeit und Relevanz der Fragen statt. Alle Patienten wurden über die Studie informiert und gaben ihr schriftliches Einverständnis. Ein positives Ethikvotum lag für den Feldtest (Nummer 2010-231-f-S) vor.

### Validierung der deutschen Übersetzung des PsAQoL

#### Patienten.

Die deutsche Übersetzung des PsAQoL wurde in einer repräsentativen Kohorte von PsA-Patienten, die neben der klinischen Diagnose auch die CASPAR-Klassifikationskriterien erfüllten, untersucht [21]. Die Studienpopulation bestand aus stationären Patienten des Rheumazentrums Ruhrgebiet und ambulanten Patienten aus 4 rheumatologischen Kooperationspraxen. Patienten ohne Deutschkenntnisse oder mit kognitiven oder psychischen Komorbiditäten, die eine zuverlässige Beantwortung der Fragen verhindern konnten, wurden ausgeschlossen. Alle Patienten wurden über die Studie informiert und gaben ihr schriftliches Einverständnis. Die Untersuchung der Patienten erfolgte als Baseline-Visite in Woche 0 und bei Patienten mit stabilem Krankheitsverlauf zur Testung der Zuverlässigkeit auch in Woche 2. Die Fragebögen wurden den Patienten als Papierversion zur Verfügung gestellt. Zur zweiten Visite wurden die Fragen vom Patienten zu Hause alleine beantwortet. Zur Testung der Zuverlässigkeit wurden nur Patienten mit einem stabilen Krankheitsverlauf eingeschlossen. Ein stabiler Krankheitsverlauf wurde angenommen, wenn die Patienten die Frage „Sind bei Ihnen in den letzten 2 Wochen Verschlechterungen bzw. Verbesserungen der Erkrankung aufgetreten?“ mit „nein“ beantworteten. Ein positives Ethikvotum lag für die Validierung (Nummer 2011-238-f-S) vor.

#### Datenerhebung.

Neben demografischen und klinischen Variablen inklusive der laufenden medikamentösen Therapie beantworteten die Patienten neben einem Schmerzfragebogen und dem Patient Acceptable Symptom State (PASS) folgende Fragebögen: deutsche Version des PsAQoL, des generische Nottingham Health Profile(NHP)-Fragebogens sowie des Health Assessment Questionnaire (HAQ).

Der Schmerz wurde mit der numerischen Ratingskala (NRS) für Schmerzen von 0 bis 10 erhoben, wobei mit 10 der maximal vorstellbare Schmerz angegeben wird.

Der „Patient Acceptable Symptom State“ (PASS) besteht aus der Frage „Vorausgesetzt Sie bleiben in dem jetzigen Zustand für die nächsten Monate, glauben Sie, dass ihr Zustand akzeptabel ist?“, die mit ja oder nein beantwortet werden kann. Das Konzept des PASS definiert als kategorialen Endpunkt diejenige Symptomstufe, an der der Patient seinen Krankheitsstatus selbst als akzeptabel einstuft [22].

Der aus 38 Einzelfragen bestehende NHP erfasst den subjektiven Gesundheitszustand in 6 Domänen: Energieverlust, Schmerz, emotionale Reaktion, Schlaf, soziale Isolation und physische Mobilität [23]. Für jede Domäne wird die positive Zustimmung zu der Frage als Prozentsatz kalkuliert. Der Wertehorizont liegt zwischen 0 und 100 %, wobei höhere Werte einen eingeschränkten Gesundheitszustand bedeuten.

Der HAQ, erfasst als Selbstauskunftsbogen die körperliche Funktionsfähigkeit. Der HAQ besteht aus insgesamt 20 Fragen, die in 8 Funktionskategorien unterteilt sind: Ankleiden, Aufstehen, Essen, Gehen, Körperpflege, Gegenstände reichen, Greifen und Alltagstätigkeiten [24]. Der Patient bewertet auf einer Skala von 0 bis 3, ob er die Tätigkeiten ohne Schwierigkeiten verrichten (Skalenwert 0, Minimumwert) oder gar nicht verrichten kann (Skalenwert 3, Maximalwert). Der HAQ-Summenscore errechnet sich als Mittelwert der Einzelaussagen, wobei ein höherer Wert auf eine schlechtere Funktionsfähigkeit hinweist. Angaben zu Verwendung von Hilfsmitteln und Fremdhilfebedarf wurden mithilfe des HAQs erhoben.

#### Statistik.

Zur Beschreibung der Kohorte wurden deskriptive Methoden verwendet. Zur Erfassung der psychometrischen Merkmale des PsAQoLs wurden folgende Analysen durchgeführt: Die Verteilung des PsAQoL-Summenwertes wurde genutzt, um Boden- und Deckeneffekte des Fragebogens zu entdecken (% der Patienten, die den minimalen oder maximalen Summenwert aufweisen). Die Konstruktvalidität wurde mittels Spearman-Korrelationskoeffizient zwischen dem PsAQoL-Summenwert und anderen klinischen Endpunkten berechnet (HAQ, NHP). Es wurde a priori die Hypothese einer moderaten, guten bzw. sehr guten Korrelation durch einen Korrelationskoeffizienten im Bereich von 0,5–0,69 bzw. 0,7–0,84 und ≥0,85 definiert. Die interne Konsistenz wurde mittels Cronbach’s α Koeffizient berechnet. Eine adäquate interne Konsistenz liegt bei einem Cronbach’s α Koeffizienten von ≥0,70 vor. Die Test/Retest-Reliabilität wurde mittels Spearman-Korrelationskoeffizient berechnet. Zur Bestätigung einer Test/Retest-Reliabilität war ein Korrelationskoeffizient mit einem Schwellenwert von ≥0,85 erforderlich [14]. Die Untersuchung der Gruppenvalidität erfolgte durch Berechnung der PsAQoL-Mittelwerte für vordefinierte Statusgruppen: Beschäftigungsstatus (berufstätig, nicht berufstätig), Nutzung von Hilfsmitteln (ja/nein), PASS (ja/nein) und Schmerz (Kategorie wenig Schmerzen [NRS 0–5] vs. viel Schmerzen [NRS ≥6]). Nichtparametrische Tests für unabhängige Stichproben (Mann-Whitney-*U*-Test für 2 Gruppen oder Kruskal-Wallis-Einweg-Varianzanalyse für 3 oder mehr Gruppen) wurden verwendet, um die Unterschiede im PsAQoL zwischen den Gruppen zu testen. Ein *p*-Wert <0,05 wurde als signifikant angenommen. Die statistischen Analysen wurden mittels „Statistical Package for Social Science“ (SPSS; IBM SPSS Statistics for Windows, Version 19.0., IBM Corp., Armonk, NY, USA) Version 19.0. durchgeführt.

## Ergebnisse

### Übersetzung des PsAQoL-Fragebogens

Die Übersetzung wurde durch ein bilinguales Team (Englisch/Deutsch) bestehend aus 5 Personen mit der Muttersprache Deutsch durchgeführt. Das Gremium erörterte Übersetzungsvorschläge, die bis zum Erreichen einer Einigung diskutiert wurden. Nach Einigung auf eine finale Version wurde die Übersetzung dem Laiengremium vorgelegt. Das Laiengremium bestand aus 3 Männern und 4 Frauen im Alter von 19 bis 68 Jahren mit durchschnittlichem Bildungsniveau (Zivildienstleistender, Arbeiter, Auszubildende, Hausmeister, Rentnerin, Telefonistin, Rezeptionistin), die ebenfalls die Übersetzungsvorschläge erörterten. Das Laiengremium hielt das Sprachniveau für adäquat und verständlich. Die Formulierungen 3, 6, 12, 18 und 19 wurden intensiv diskutiert. Die 6. Aussage „I often get angry with myself“ wurde initial als „Ich ärgere mich oft über mich selbst“ übersetzt; dann aber in „Ich bin häufig unzufrieden mit mir“ geändert, da im Gremium Einigkeit herrschte, dass man sich zwar über Fehler aufregen kann, aber nicht über sich selbst. Die 3. Aussage „Es ist mir zu anstrengend auszugehen“ wurde mit „… und Leute zu treffen“ ergänzt, da betont wurde, dass eine Person auch ausgehen kann, ohne andere Menschen zu treffen (z. B. ins Museum), dies aber nicht dem englischen Originalsatz „It’s too much effort to go out and see people“ entsprechen würde. Die initiale Übersetzung der Aussage 12 „Ich bin leicht aus der Fassung zu bringen“ wurde in „Ich bin durch andere Menschen schnell gereizt“ geändert. In der Aussage 19 wurde das Wort „Verrichtung“ durch „tägliche Aufgaben“ ersetzt, da das Wort „Verrichtung“ nicht als allgemeingebräuchlich angesehen wurde. Es fanden keine Veränderungen im Wortlaut der Anweisung und der Danksagung statt.

### Testung der Übersetzung in einem Feldtest

Im Juni/Juli 2010 nahmen insgesamt 10 Patienten mit PsA (4 Männer und 6 Frauen, Alter 52,5 ± 10,4 Jahre) aus dem Rheumazentrum Ruhrgebiet an dem Feldtest teil. Die mittlere Zeitdauer für die Beantwortung der Fragen des PsAQoL lag bei 4,7 ± 2,1 min mit einer Spannbreite zwischen 2 und 8 min. Der Summenwert des PsAQoL lag im Mittel bei 8,9 ± 5,2 mit einer Spannbreite zwischen 2 und 16. Die Teilnehmer des Feldtestes empfanden die Fragen generell als leicht verständlich und angemessen sowie als adäquat und klar formuliert. Die 20 Aussagen decken die Bandbreite der verschiedenen Aspekte der Lebensqualität ab. Die Aussagen 2, 6, 7 und 8 wurden intensiv diskutiert: Für Aussage 2 (Körperpflege) wurde kritisch hinterfragt, wie umfangreich eine gründliche Körperpflege definiert ist, in Aussage 6 (Unzufriedenheit mit sich selber) kritisiert, dass die Aussage sich nicht explizit auf die Krankheit an sich beziehen würde. Für die Aussage 7 (eigene Ziele) wurde lange über die korrekte Wortwahl („das“ oder „alles“) diskutiert, zu der Aussage 8 (Wahrnehmung des eigenen Alters) wurde angemerkt, dass das Konzept nicht krankheitsspezifisch sei. Die Aussagen 9 und 10 wurden als inhaltlich redundant angesehen, da in beiden Aussagen die Limitation der Freizeitgestaltung angesprochen würde. Drei Patienten gaben an, dass der PsAQoL-Fragebogen zu sehr auf „psychologischen Stress“ ausgerichtet ist, dagegen merkte 1 Patient an, dass sich der Fragebogen nicht ausführlich genug auf psychologische Probleme, wie z. B. einen Suizidversuch, bezieht. Jeweils 1 Patient wünschte sich, dass im Fragebogen das Thema „Angst vor Medikation“ bzw. „Hautbeteiligung bei Psoriasis“ Eingang gefunden hätte. Eine Patientin bemängelte die schwache Gewichtung der Domäne Erwerbsfähigkeit. Drei Patienten wünschten sich eine Antwortoption mit 3 anstatt 2 Antwortmöglichkeiten: nicht zutreffend, manchmal zutreffend und zutreffend.

### Validierung

Insgesamt wurden 126 Patienten mit PsA von 2011 bis 2012 eingeschlossen. Die Kohorte bestand aus Patienten mit einer überwiegend peripheren Arthritis (84), gefolgt von Patienten mit gemischten (29) bzw. rein axialen Manifestationen (12). Ein Patient ließ diese Frage unbeantwortet. Insgesamt 44 Patienten hatten eine kontinuierliche und 40 eine bedarfsadaptierte Therapie mit nichtsteroidalen Antirheumatika (NSAR). Eine dauerhafte Glukokortikoid-Therapie hatten 60 Patienten. Die mittlere Prednisolon-Dosis lag bei 3,9 mg ± 6,61 mg. Eine konventionelle (csDMARD) Basistherapie erhielten 97 Patienten (Methotrexat [*n* = 67], Leflunomid [*n* = 10], Sulfasalazin [*n* = 9] und andere csDMARDs [*n* = 10]). Eine bDMARD-Therapie mit einem TNF-α-Inhibitor bekamen 18 Patienten. Ein zusätzliches Opioidpräparat erhielten 14 Patienten. Die demografischen und klinischen Charakteristika der Kohorte zu Baseline (Woche 0) sind in Tab. 1 dargestellt.

**Table 1:** 

Charakteristika	Ergebnis (*n* = 126)
*Alter* (Jahre), MW (SD), Spannbreite	55,6 (11,3), 21–77
*Männliches Geschlecht, n* (%)	47 (37,3)
*Symptomdauer* (Jahre), MW (SD)	9,8 (11,06)
*Ausbildungsdauer, n* (%), *n* = 124	
<8 Jahre	31 (25,0)
8 bis 12 Jahre	72 (58,1)
>12 Jahre	21 (16,9)
*Erwerbstätigkeit, n* (%)	
Vollzeit	37 (29,4)
Teilzeit	23 (18,3)
Rentner	40 (31,7)
Ausbildung	3 (2,4)
Erwerbsminderungsrente	7 (5,6)
Hausfrau/Hausmann	16 (12,7)
*PsAQoL*, MW (SD)	8,7 (5,9)
*PASS mit ja beantwortet, n* (%)	56 (45,5)
*Schmerz* (NRS 0–10), MW (SD)	4,6 (2,6)
*HAQ Total*, MW (SD)	0,9 (0,7)
*NHP*, MW (SD)	
Energieverlust, *n* = 124	54,8 (39,5)
Schmerz, *n* = 121	51,4 (36,9)
Emotionale Reaktion, *n* = 123	25,2 (28,3)
Schlafstörungen, *n* = 125	39,7 (31,1)
Soziale Isolation, *n* = 123	11,4 (24,8)
Physische Mobilität, *n* = 125	37,3 (26,5)

*MW* Mittelwert, *SD* Standardabweichung, *PASS* „patient acceptable symptomatic state“, *NSAR* nichtsteroidale Antirheumatika, *MTX* Methotrexat, *DMARD* „disease modifying anti-rheumatic drugs“, *PsAQoL* „psoriatic arthritis quality of life“

Es wurden 68 Patienten, die keine Änderung der Krankheitsaktivität nach 2 Wochen angaben, zur Berechnung der Test/Retest-Reliabilität in den Zuverlässigkeitsarm eingeschlossen.

#### Psychometrische Eigenschaften des PsAQoL

**Figure 1:**
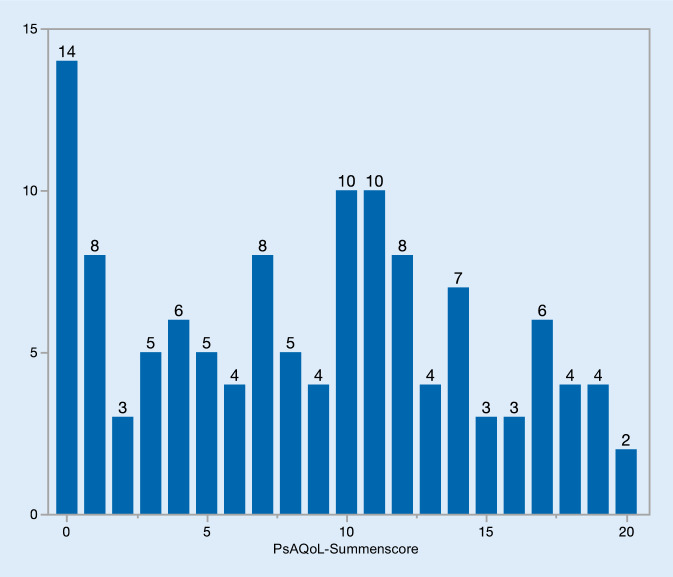

Der PsAQoL wies im Mittel einen Wert von 8,7 ± 5,9 mit einer Schwankungsbreite von 0 bis 20 in der Woche 0 auf. Bei 14 Patienten (11,4 %) fand sich ein Bodeneffekt (Summenwert von 0). Die 14 Patienten hatten eine niedrige Krankheitsaktivität: NRS Schmerz zwischen 0 und 3, davon gaben 4 Patienten (28,5 %) einen NRS Schmerz von 0, 4 Patienten (28,5 %) einen NRS Schmerz von 1, 3 Patienten (21,4 %) einen NRS Schmerz von 2 und 3 Patienten (21,4 %) einen NRS Schmerz von 3 an; 50 % waren Männer, die Krankheitsdauer variierte zwischen 0 und 27 Jahren. Alle Patienten beantworteten die Frage des PASS-Fragebogens mit „ja“. Bei 2 Patienten (1,6 %) fand sich ein Deckeneffekt (Summenwert von 20) (Abb. 1).

Konstruktvalidität: Es zeigte sich eine gute Korrelation des PsAQoL-Fragebogens mit den NHP-Subdomänen: Energieverlust (0,75), Schmerz (0,70), emotionale Reaktion (0,75), physische Mobilität (0,70) in der Woche 0, was die Bedeutung dieser Dimensionen für die Lebensqualität widerspiegelt (Tab. 2).

**Table 2:** 

Assessment	Spearman‐Korrelation
*NHP*	
Energieverlust Skala	0,75**
Schmerz Skala	0,70**
Emotionale Reaktion	0,75**
Schlaf Skala	0,58**
Soziale Isolation	0,60**
Physische Mobilität	0,70**
*HAQ*	
Total	0,65**

Eine moderate Korrelation zeigte sich zwischen PsAQoL und HAQ (0,65) sowie den NHP-Subdomänen Schlaf und soziale Isolation (Tab. 2).

##### Interne Konsistenz.

Die interne Konsistenz für den PsAQoL war mit einem Cronbach’s α Koeffizienten von 0,92 zur Baseline und 0,95 zur Woche 2 hoch.

##### Test/Retest-Zuverlässigkeit.

Insgesamt wurden Fragebögen von 68 Patienten mit stabilem Krankheitsverlauf zur Messung der Zuverlässigkeit (Reliabilität) nach 2 Wochen analysiert. Der PsAQoL-Summenwert in dem Zuverlässigkeitsarm lag bei Baseline bei 7,7 ± 6,0 und zur zweiten Visite nach 2 Wochen bei 7,4 ± 6,5. Es lag eine sehr gute Zuverlässigkeit mit einem Spearman-Korrelationskoeffizienten von 0,94 vor.

##### Gruppenvalidität.

Es zeigte sich erwartungsgemäß ein signifikanter Unterschied im PsAQoL-Summenscore bei berufstätigen vs. nicht berufstätigen Patienten, bei Patienten mit und ohne Nutzung von Hilfsmitteln sowie bei Patienten mit und ohne viel Schmerzen und nach Angaben im PASS-Fragebogen (Tab. 3).

**Table 3:** 

	Anzahl	PsAQoL*
*Beschäftigungsstatus*		
Berufstätig	61	7,4 (5,7)
Nicht berufstätig	62	9,9 (5,9)
*Signifikanzniveau*	123	<0,05
*Hilfsmittel*		
Nein	76	7,1 (5,8)
Ja	47	11,4 (5,0)
*Signifikanzniveau*	123	<0,05
*PASS*		
Ja	54	4,4 (4,4)
Nein	66	12,2 (4,4)
*Signifikanzniveau*	120	<0,05
*Schmerz*		
0–5	77	6,5 (5,4)
6–10	45	12,7 (4,4)
*Signifikanzniveau*	122	<0,05

## Diskussion

Der PsAQoL wurde erfolgreich ins Deutsche übersetzt, und die deutsche Version konnte anschließend adäquat validiert werden. Wie in den qualitativen Patienteninterviews gezeigt werden konnte, liegt eine hohe Augenschein- und Inhaltsvalidität des Fragebogens vor. Das Ziel des Feldtestes, die Überprüfung der Übersetzung hinsichtlich Anwendbarkeit, Verständlichkeit und Relevanz als auch der Akzeptanz des Fragebogens bei den Patienten, konnte überzeugend erreicht werden. Die psychometrischen Eigenschaften der deutschen Version des PsAQoLs sind insgesamt gut und belegen die Validität und Zuverlässigkeit des Fragebogens. Der Bodeneffekt war akzeptabel, wobei fast kein Deckeneffekt beobachtet wurde. Die Konstruktvalidität zeigte eine gute Korrelation mit relevanten Domänen der Lebensqualität. Der PsAQoL korrelierte moderat mit der körperlichen Funktionsfähigkeit, gemessen mit dem HAQ (0,65), und gut mit den 4 Domänen des NHP-Fragebogens zur Lebensqualität. Die moderate Korrelation des PsAQoL mit dem HAQ spiegelt wider, dass dieses Instrument v. a. auf körperliche Funktionsfähigkeit fokussiert und damit nur eine Facette der Erkrankung erfasst.

Da der PsAQoL nur 20 Fragen mit einer dichotomen Antwortoption umfasst, ist das Ausfüllen des Fragebogens auch in der klinischen Routine durchführbar. Der Fragebogen war mit einer durchschnittlichen Ausfüllzeit von weniger als 5 min einfach zu vervollständigen. Damit liegt erstmals ein krankheitsspezifisches Messinstrument zur Erfassung der HRQoL von deutschsprachigen Patienten mit PsA vor.

Die Testeigenschaften der deutschen Übersetzung des PsAQoLs stehen im Einklang mit der originalen englischen Version sowie mit Übersetzungen ins Schwedische, Niederländische, Portugiesische und Griechische, bei denen ebenfalls robuste psychometrische Testeigenschaften gezeigt werden konnten [14, 16–18, 20].

Bodeneffekte im NHP und HAQ waren höher, das spiegelt wider, dass der PsAQoL zur Erfassung der Lebensqualität zu bevorzugen ist.

Eine Limitierung unserer Studie ist, dass die Änderungssensitivität des deutschen Fragebogens nicht untersucht wurde. Es gibt jedoch zunehmend mehr Daten zur Änderungssensitivität des PsAQoL. In einer kleinen Studie aus dem Jahr 2008 mit 28 englischsprachigen Patienten wurde erstmalig die Änderung im PsAQoL nach DMARD-Therapieumstellung mittels „standardized response mean“ (SRM) untersucht [25]. Die Effektstärke war nach 3 Monaten groß (SRM −0,71) und nach 6 Monaten klein (SRM −0,41). Diese Studie schloss allerdings eine kleine Zahl von Patienten ein und wurde nicht placebokontrolliert durchgeführt. In einer 2014 publizierten randomisierten placebokontrollierten internationalen Studie (*n* = 409) zeigte sich nach 24 Wochen eine deutliche Verbesserung der mit dem PsAQoL gemessenen Lebensqualität bei den mit Certolizumab (CTZ) behandelten PsA-Patienten im Vergleich zu Placebo. Bereits nach 4 Wochen war ein signifikanter Unterschied im PsAQoL-Summenscore nachweisbar: in der Placebogruppe −0,8 ± 3,7 vs. −2,4 ± 3,9 bei mit CTZ behandelten Patienten (CTZ alle 2 Wochen) bzw. −2,5 ± 4,5 (CTZ alle 4 Wochen) [26]. All diese Daten dokumentieren eine ausreichende Änderungssensitivität. Zudem wurde der PsAQoL-Fragebogen als Messinstrument mit guter Validität und Zuverlässigkeit von GRAPPA/OMERACT 2018 in einem systematischen Review über psychometrische Eigenschaften von verschiedenen Patient Reported Outcomes (PRO) bei PsA bewertet [11].

Zusammenfassend liegt mit der deutschen Version des PsAQoL-Fragebogens ein valides und zuverlässiges krankheitsspezifisches Messinstrument zur Erfassung der HRQoL bei Patienten mit PsA vor. Der Fragebogen ist verständlich und leicht auszufüllen, sodass er für die Anwendung im klinischen Alltag und in der klinischen Forschung geeignet ist.
